# Mitochondria-Associated Endoplasmic Reticulum Membranes (MAMs) and Their Prospective Roles in Kidney Disease

**DOI:** 10.1155/2020/3120539

**Published:** 2020-09-03

**Authors:** Peng Gao, Wenxia Yang, Lin Sun

**Affiliations:** ^1^Department of Nephrology, The Second Xiangya Hospital, Central South University, Changsha, Hunan, 410011, China; ^2^Key Laboratory of Kidney Disease & Blood Purification, in Hunan Province, Changsha, Hunan, 410011, China; ^3^Institute of Nephrology, Central South University, Changsha, Hunan, 410011, China

## Abstract

Mitochondria-associated endoplasmic reticulum (ER) membranes (MAMs) serve as essential hubs for interorganelle communication in eukaryotic cells and play multifunctional roles in various biological pathways. A defect in ER-mitochondria signaling or MAMs dysfunction has pleiotropic effects on a variety of intracellular events, which results in disturbances of the mitochondrial quality control system, Ca^2+^ dyshomeostasis, apoptosis, ER stress, and inflammasome activation, which all contribute to the onset and progression of kidney disease. Here, we review the structure and molecular compositions of MAMs as well as the experimental methods used to study these interorganellar contact sites. We will specifically summarize the downstream signaling pathways regulated by MAMs, mainly focusing on mitochondrial quality control, oxidative stress, ER-mitochondria Ca^2+^ crosstalk, apoptosis, inflammasome activation, and ER stress. Finally, we will discuss how alterations in MAMs integrity contribute to the pathogenesis of kidney disease and offer directions for future research.

## 1. Introduction

Diabetic kidney disease (DKD) is a major microvascular complication of diabetes and the leading cause of end stage renal disease (ESRD) in the United States and China [1, 2]. Although kidney replacement treatments such as dialysis and renal transplantation have offered proven efficacy for patients with ESRD, there is still a large residual risk of DKD onset and progression [3]. In addition, kidney disease shows the strongest correlation with mortality in patients with diabetes compared with other vascular complications [4]. Thus, the renal tissue is an important target of hyperglycemic damage, but the mechanisms underlying this damage are not fully understood.

Several scenarios have been postulated for DKD. In addition to inflammation, perturbations in mitochondrial quality control and ER stress have been acknowledged as major factors governing the development and progression of DKD [5, 6]. While the impacts of ER and mitochondrial dysfunction in DKD have largely been viewed and studied independently, the mitochondria and ER membranes physically interact with each other at specific subdomains termed mitochondrion-associated endoplasmic reticulum membranes (MAMs) that are functionally involved in the modulation of interorganellar Ca^2+^ exchange [7, 8], mitochondrial dynamics [9], inflammasome assembly [10], activation of autophagy/mitophagy [11–13], redox signaling control [14], and ER stress [15], and each of these processes has been reported to be involved in the occurrence and progression of DKD. These facts indicate that changes in MAMs may underlie many of the phenotypes that control the pathological progression of kidney disease. However, there are currently few studies focusing on MAMs' involvement in kidney disease by far (see Table 1), especially in the beginning, much of this work was merely descriptive. In view of this, this review is aimed at filling the gap towards kidney diseases to lay a foundation for future studies building on existing data.

## 2. MAMs: Structure and Composition

The existence of MAMs was first discovered in the1950s by electron microscopy [16, 17], and they were biochemically identified in the 1990s [18]. Csordás et al., using limited proteolysis, firstly confirmed that the connection between the ER and the mitochondria is composed of proteinaceous tethers [19]. Thus far, the use of in-depth mass spectrometry analysis in tissues such as the mouse brain [20], liver [21], and testes and human testes [22] has identified various proteins (approximately 1000) that reside within the MAMs and might help to advance this field. According to these results, one could conclude that the components of MAMs were highly conserved not only between in species (human and mouse) but also between different tissues (brain and testes). It is noteworthy that the above proteomic analyses were performed with tissues under normal conditions. Another more recent study presented a comprehensive proteome profiling of brain MAMs isolated from db/db (mice with genetic defects in the leptin receptor which recapitulates many clinical features of human type 2 diabetes mellitus) and db/m (nondiabetic control) mice, among which 144 proteins were found to be significantly altered by chronic hyperglycemia [23], suggesting that the composition of MAMs was significantly modified under diabetic states. Based on these structural and component characteristics, MAMs are thought to be highly flexible and plastic structures capable of recruiting a variety of signaling molecules according to the a cell's needs [24].

**Figure 1:**
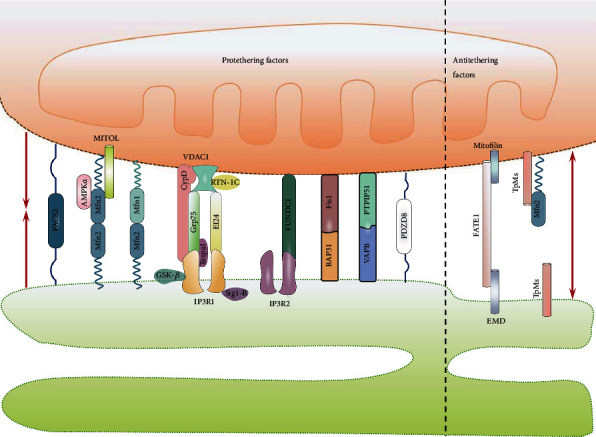


A core subset of proteins that tether mitochondria to the ER in mammalian cells has been identified; these proteins either play a direct role in the physical connection of MAMs or modulate the tethering complexes in MAMs (Figure 1). The well-established proteins include (1) the protethering complexes or factors: (i) the phosphor acidic cluster sorting protein 2 (PACS2) [15], (ii) glucose-regulated protein 75 (Grp75) bridging inositol triphosphate receptor (IP3R) to voltage-dependent anion channel 1 (VDAC1) [25], (iii) mitofusin 2 (Mfn2) on the ER that bridges the two organelles by engaging in homotypic and heterotypic complexes with Mfn 1 or 2 on the outer mitochondrial membrane (OMM) [26], (iv) the mitochondrial fission 1 protein- (Fis1-) B cell receptor-associated protein 31 (BAP31) complex (ARCosome) [27], (v) the complex formed by vesicle-associated membrane protein-associated protein B (VAPB) and protein tyrosine phosphatase interacting protein 51 (PTPIP51) [28, 29], (vi) the FUN14 domain containing 1- (FUNDC1-) IP3R2 complex [30], (vii) PDZD8 [31], (viii) Beclin1 (BECN1) [13], and (ix) MITOL, Parkin, and AMPK*α*, which regulate MAMs formation by directly interacting with mfn2 on the OMM side [32, 33]; (2) the proteins that modulate IP3Rs/Grp75/VDAC complexes: Sigma-1 receptor (Sig-1R) [34], cyclophilin D (CypD) [35], thymocyte-expressed, positive selection-associated gene 1 (Tespa1) [36], reticulon 1C (RTN-1C) [37], glycogen synthase kinase-3*β* (GSK3*β*) [38], disrupted-in-schizophrenia 1 (DISC1) [39], mitochondrial translocase of the outer membrane 70 (TOM70) [40], transglutaminase type 2 (TGM2) [41], Wolfram syndrome 1 (WFS1) [42], pyruvate dehydrogenase kinases 4 (PDK4) [43], and etoposide-induced protein 2.4 (EI24) [44]; (3) antitethering factors: (i) trichoplein/mitostatin (TpMs) that negatively regulates MAMs tethering via Mfn2 [45], (ii) FATE1 uncoupling MAMs by interacting with ER chaperones and emerin (EMD) and the mitofilin [46], and (iii) Caveolin-1 [47]; 4) Upstream regulators of MAMs formation: (i) glycogen synthase kinase-3*β* (GSK3*β*) [28], (ii) p38 MAPK [48], (iii) cGMP-dependent protein kinase (PKG) [49], (iv) FOXO1 [43], (v) cAMP-dependent protein kinase (PKA) [47], and (vi) AMPK*α* [32, 50]. The functional roles and relative protein expression of MAMs-resident proteins listed in this text are summarized in Table 2. However, these sites of “contact” with organelles are not truly touching each other. The distance between the ER and the OMM ranges from 10 to 80 nm, ~10 and ~25 nm across at the smooth ER (sER), and ~50 to ~80 nm at the rough ER (rER) [51]. On the other hand, the length of MAMs covers 4 to 20% of the total mitochondrial surface, depending on its cellular stress and metabolic state [7, 52]. Thus, the number, length, and width of the contact zones should be considered important parameters to control and define the biological functions of MAMs.

## 3. MAMs: Assessment and Manipulation

**Figure 2:**
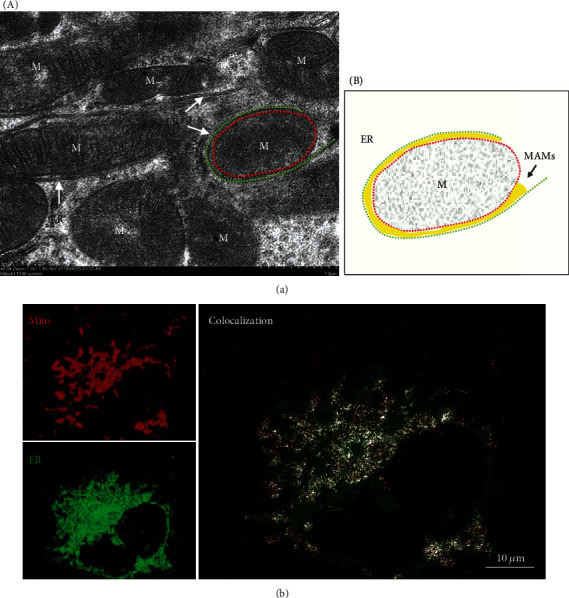


Many approaches have been used to evaluate the structure and function of MAMs. The most preeminent methodology to visualize MAMs interactions is transmission electron microscopy (TEM), which offers the nanometer resolution required to quantify the thickness of MAMs, the number of contacts, and the length of their interface [19] (Figure 2(a)). This technique, coupled with thin tomography, has been used to provide further insight into the 3D nature of organelle contacts [19, 53]. However, TEM is labor intensive and can reflect only the static status of the cells. More importantly, it cannot reveal true MAMs but rather only physical proximity; TEM can reveal a mitochondrion surrounded by an ER, but physical proximity is not necessarily a MAM (Figure 2(a), B). In fact, according to the definition of MAMs, such a structure may contain MAMs areas but does not represent a MAM around the entire mitochondrion. Without labeling of the respective MAMs-associated proteins, one can only claim to visualize physical proximity but not MAMs in the given figure. Special care should be taken to analyze experimental results when using TEM to quantify MAMs. Confocal microscopy is another strategy to measure juxtaposition between two organelles by using the mitochondria and ER selectively targeted fluorescent proteins (FPs) [26] or Tracker probes [23] (Figure 2(b)), and Manders' colocalization coefficient calculated by ImageJ is used to measure the colocalization of ER and mitochondria [26]. In general, MitoTracker probe accumulation and labeling the organelle is dependent on the mitochondrial membrane potential. Thus, the use of mitochondrial-targeted genetically encoded FPs, which are not sensitive to changes of mitochondrial conditions, including membrane potential and redox status, is highly recommended [54]. Additionally, alterations in organelle morphology could complicate the interpretation of fluorescence imaging and can sometimes be misleading [55]. More importantly, the thickness of MAMs is far below the resolution of confocal microscopes (approximately 150-300 nm). Therefore, this approach is more a measure of organelle proximity rather than the formation of actual MAMs [53, 55]. Even so, it is the easiest and most widely available approach to analyze the dynamic of MAMs in living cells. To overcome these drawbacks, a split-GFP-based reporter was designed to monitor dynamic changes in MAMs by confocal microscopy under normal or stressful cellular conditions [56]. Recently, an *in situ* proximity ligation assay (PLA) has been applied to visualize and quantify endogenous MAMs in fixed cells by using the close proximity between proteins of the OMM (VDAC1) and of the ER membrane (IP3R1/2) at the MAMs interface [57, 58]. Finally, due to their specific lipid nature that renders them lower density membranes, MAMs can then be purified using Percoll density gradients and ultracentrifugation [59, 60]. A biochemically isolated MAMs fraction consists of membrane fragments from both the ER and the OMM that had been in close contact at the time of cellular subfractionation. Besides, the ER portion of the MAMs fraction has the features of a detergent-resistant lipid raft and is rich in cholesterol and sphingomyelin [61–63]. While there is still a lack of appropriate markers to detect MAMs, commonly used markers include long-chain acyl-CoA synthase (ACS4/FACL4) [64], phosphatidylethanolamine N-methyltransferase 2 (PEMT2) [65], phosphatidylserine synthases (PSS1/2), and diacylglycerol acyltransferase (DGAT2) [66]. Among them, FACL4 is thought to be the most reliable protein. However, it should be noted that none of the above proteins are exclusively presented at MAMs. Therefore, combined detection of MAMs-rich proteins (such as FACL4) along with other organelle-specific markers (i.e., ER, mitochondria, nucleus, and cytosol) is highly recommended to characterize a good fractionation of MAMs [59]. Additionally, MAMs can still be isolated from frozen samples, although these should only be used for protein or lipid composition analysis. If needed for any analysis of activity, MAMs should be isolated from fresh samples and assayed within 2-3 h after isolation [60].

Finally, elucidation of the integrity of MAMs is required for many aspects of cell function in mammalian cells, and several strategies have been devised to attain experimental manipulation of MAMs. To artificially both tighten and expand the connections of MAMs, a constitutive linker consisting of ER and OMM targeting sequences (Sac1 or yUBC6 and mAKAP1, respectively) connected through a fluorescent protein (RFP) with a length of ~15 nm has proven useful both *in vitro* [19] and *in vivo* [67]. To tighten the preexisting physical coupling of MAMs, a drug inducible linker has been developed, which consists of an OMM-targeted FKBP (FK506 binding protein) and an ER-targeted FRB (FKBP12-rapamycin binding domain). The addition of rapamycin causes heterodimerization between the adjacent FKBP and FRB domains to rapidly tighten interorganellar contacts [68], and this connection usually lasts 1 hour, even after rapamycin removal [39]. Despite this approach being both useful and elegant, it should be applied with caution, especially when examining autophagy, as rapamycin is a potent autophagy inducer, and autophagosomes can form at MAMs [11]. Additionally, rapamycin binds to endogenous FKBP12, creating a trimolecular complex with mTOR and leading to the inhibition of mTORC1, a central regulator of various cellular processes, including autophagy, ribosome biogenesis, protein synthesis and turnover, and the metabolism of lipids, nucleotides, and glucose [69]. In some cases, prolonged treatment with rapamycin can also indirectly inhibit the mTORC2 complex [69], which would make the experimental results more complicated. In light of this, rapamycin should therefore only be applied for a short time (2~10 min) and at the lowest concentration (100~150 nM) to minimize the broad effects initiated by its endogenous receptors [39, 68, 70].

Genetic or pharmacological manipulation of MAMs-resident proteins is a good approach to modulate the formation of MAMs, and these tethering proteins include but are not limited to Sig-1R [71], Grp75 [70, 72, 73], Mfn2 [35, 58, 70], FUNDC1 [35, 50], PACS2 [67], and IP3R1 [67]. Overexpression or silencing of the above relevant proteins results in increases or decreases in MAMs formation, respectively. Special care should be taken when selecting these candidate proteins, since none of these proteins are exclusively expressed at MAMs, and their manipulation may result in alterations of cellular functions outside of MAMs. For instance, PACS2 and Mfn2 also affect the morphology of the ER and mitochondria, which would complicate the interpretation of such experiments [15, 26]. To some extent, these complicated dual effects also explain the existence of many apparently contradictory results and potentially controversial issues in this field. Regarding strategies used to disrupt MAMs, manipulation of FATE1 expression might be the preferred alternative, because FATE1 expression is mainly restricted to the testis, adrenal gland, and in a variety of cancers [46]. FATE1 overexpression has also been successfully performed to reduce MAMs in human myotubes [58] and in human proximal tubular cells (HK-2) [74].

## 4. Modulation of Mitochondrial Quality Control

The mitochondrial quality control (MQC) system involves intricate regulation of mitochondrial dynamics (fission/fusion), redox balance, bioenergetics, and mitophagy [75], and all of these described processes have been closely associated with MAMs' dysfunction [76], suggesting that MAMs act as a signaling hub orchestrating these quality control mechanisms. It has also been established in humans and mouse models that disturbance of the mitochondrial quality control processes results in mitochondrial dysfunction and the progression of DKD [5].

### 4.1. Coordination of Mitochondrial Fusion/Fission

Mitochondria are a network of plastic organelles that actively undergo fusion and fission processes to optimize mitochondrial function and quality control [77]. Mitochondrial fusion allows the intermixing and exchange of the contents of partially damaged mitochondria, which is thought to counteract the decline of mitochondrial functions [77]. However, disturbances in this equilibrium, featuring a shift towards mitochondrial fission, is clearly noted in patients and experimental models with DKD [78–81], and this mitochondrial phenotype even precedes the onset of renal functional and structural changes in experimental models [82], lending support for a causal role of excessive mitochondrial fission in the development of the DKD.

**Figure 3:**
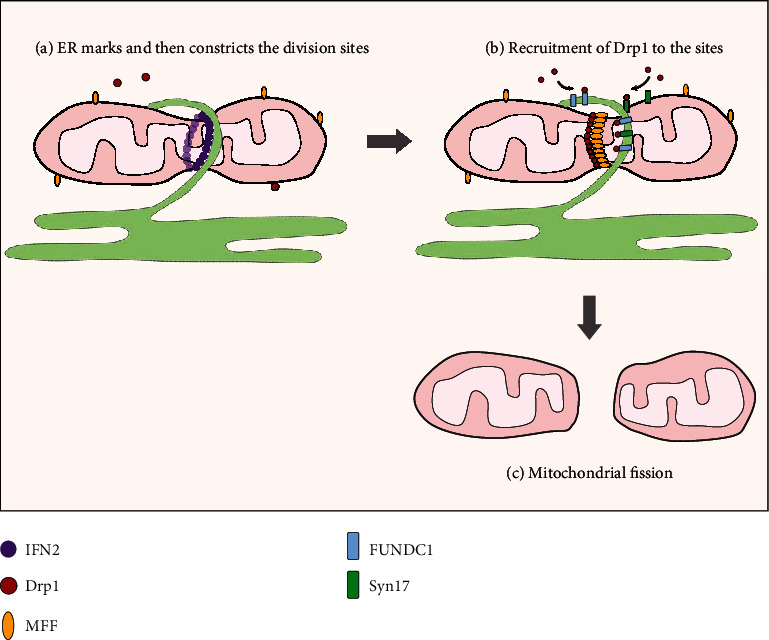


Recently, a wide area of research has provided new insights into the role of MAMs in mitochondrial dynamics [9, 83–86], indicating an additional role of MAMs in the pathogenesis of DKD. Connections with mitochondrial fusion could be based on the core components of the mitochondrial fusion machinery, Mfn1 and Mfn2, which localize to the MAMs [26]. Compared to fusion, the relevance of MAMs in mitochondrial fission has been well studied (Figure 3). Mechanistically, mitochondrial fission is conventionally governed by dynamin-related 1 (Drp1) and its adaptors including mitochondrial fission factor (Mff), Fis1, and mitochondrial dynamics proteins of 49 and 51 kDa (MiD49 and MiD51, respectively), which recruit Drp1 from the cytosol to form spirals around the mitochondria to drive membrane scission [87, 88]. Interestingly, recent research has shown that prior to Drp1 recruitment, ER-mitochondria contacts first formed and marked the positions of mitochondrial constriction and subsequently facilitated Drp1 recruitment and assembly to the constricted sites to complete fission, suggesting that MAMs play an active role in the early steps of mitochondrial fission through defining the division sites [9]. Subsequent studies have further confirmed that ER-localized inverted formin 2- (INF2-) induced actin polymerization at MAMs may serve as the impetus for initial mitochondrial constriction and division by two independent mechanisms [83, 86]: (1) actin polymerization constricts the mitochondrial tubule sufficiently to fit its diameter to that of Drp1 oligomerization, leading to OMM constriction and (2) actin polymerization resulting in MAMs formation facilitates calcium transfer from the ER to the mitochondrion, triggering IMM (inner mitochondrial membrane) constriction. These observations highlight a positive feedback between MAMs formation and mitochondrial fission. There are several proteins in addition to actin that are known to regulate Drp1 activity at MAMs. SNARE protein syntaxin 17 (Syn17) is present on raft-like structures of MAMs and promotes mitochondrial fission by determining Drp1 localization and activity in fed cells. The hairpin-like C-terminal hydrophobic domain is important for this regulation [84]. FUNDC1 is another protein that regulates hypoxia-induced mitochondrial dynamics at the MAMs. FUNDC1 is enriched at the MAMs by interacting with the ER resident protein calnexin, and it serves as a new mitochondrial receptor for Drp1 to drive mitochondrial fission in response to hypoxia [85]. Collectively, MAMs might play a pivotal role in the initiation and completion of mitochondrial fission by marking the division sites and driving the fission via pleiotropic molecular mechanisms. Accordingly, it seems promising that disruption of MAMs integrity may provide more benefits for diabetic renal damage by inhibiting malignant mitochondrial fission. Additionally, mitochondrial dysfunction is associated with excessive mitochondrial division, also increasing the intriguing possibility that the mitochondrial dysfunction in DKD could involve alterations of MAMs. This notion received support from a very recent study in which diabetes-induced chronic enrichment of MAMs in podocytes and disruption of MAMs formation by knockdown of FUNDC1 expression, a key tether for MAMs, antagonized mitochondrial dysfunction and the progression of renal damage in db/db mice [50].

### 4.2. Induction and Execution of Mitophagy

**Figure 4:**
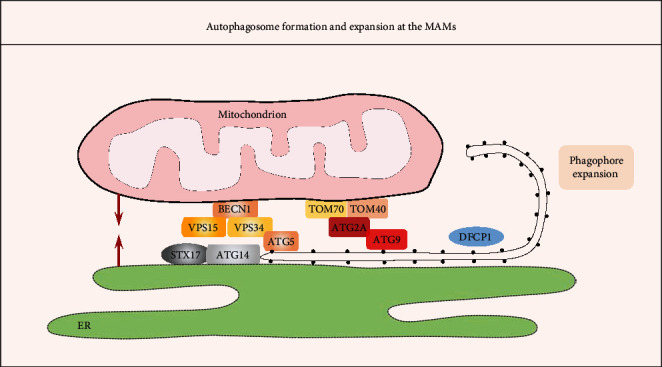


It has been proposed that MAMs are crucial for the induction and execution of autophagy based on the following supportive facts (see Figure 4): (1) Many autophagy-related gene (ATG) proteins, including ATG14 (autophagosome marker), ATG2/5 (autophagosome-formation marker), double FYVE domain-containing protein 1 (DFCP1, which serves as a platform for autophagosome formation), Beclin1, and VPS15/34, translocate to the MAMs fraction [11, 89]. (2) TOM40/70 directs Atg2A to MAMs to mediate phagophore expansion. On the MAMs, Atg2A facilitates Atg9-vesicle delivery to promote phagophore expansion and efficient autophagic flux [90]. (3) Disruption of the ER-mitochondria connection by knockdown of PACS2 or Mfn2 expression efficiently prevents the formation of ATG14 puncta [11]. Among the different forms of specialized autophagy, mitophagy is one well-studied type of selective autophagy that constitutes an important part of the MQC system by selective and timely removal of damaged and dysfunctional mitochondria [91]. Similar to autophagy, mitophagy occurs at the MAMs site both in yeast and in mammalian cells [12, 92]. Currently, 2 main pathways of mitochondrial priming for mitophagy have been suggested [91]: (1) Parkin-dependent pathways, which involve the concordance of cytosolic ubiquitin E3 ligase Parkin and the mitochondrial protein kinase Pink1 (PTEN-induced putative kinase1) and (2) adaptor-dependent mitophagy, which is mediated directly by multiple adaptor proteins, such as BNIP3 (BCL2 interacting protein 3), BNIP3L/NIX (BCL2 interacting protein 3 like), and FUNDC1, which possess an LC3 interacting region (LIR) and interact with light chain 3 (LC3) to mediate mitophagy [93]. Notably, both mitophagy pathways have also been fine-tuned by MAMs, which is discussed in greater detail below.

#### 4.2.1. Parkin/Pink1-Dependent Mitophagy

**Figure 5:**
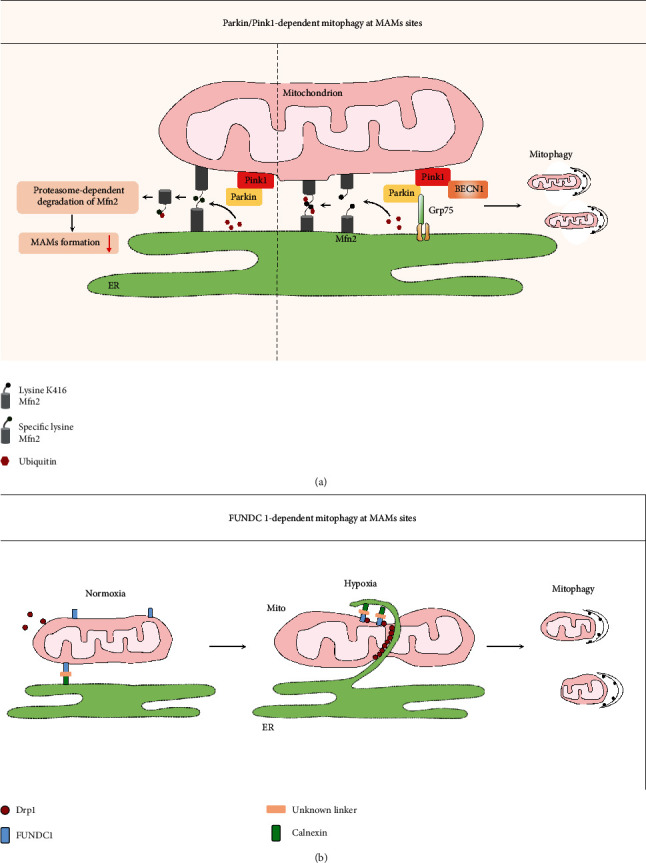


Following mitophagic stimuli, both Parkin and Beclin1 are relocalized to MAMs in a Pink1-dependent manner, and moreover, the direct interaction between Pink1 and Beclin1 enhances the formation of MAMs which is required for mitophagosome biogenesis during mitophagy [13], suggesting that Pink1 represents a master regulator of MAMs' function. However, the role of MAMs-localized Parkin in MAMs tethering and related mitophagy remains debated and partially depends on the ubiquitination sites of Mfn2. For instance, Parkin ubiquitinating Mfn2 at a specific site promotes proteasome-dependent degradation of Mfn2, consequently disrupting Mfn2-mediated MAMs tethering in primary fibroblasts [94]. However, if Parkin ubiquitinates Mfn2 on lysine K416, it would favor MAMs formation and mitophagy [95]. In addition, Parkin also favors MAMs formation possibly via interaction with Grp75 in the HeLa and SH-SY5Y cells [96]. Interestingly, neuronal excitotoxicity increases Parkin translocation to MAMs, where it promotes mitophagy only when the antioxidant NAC is present [97]. These seemingly contradictory observations suggest that the functional consequences of MAMs-relocalized Parkin could be highly cell type and/or stimulus dependent. Additional studies are required to provide a comprehensive view of how Parkin physically and functionally affects MAMs. Nevertheless, these data suggest that Parkin/Pink1 cooperates with MAMs to ensure mitophagy activation and simultaneously corrects the MAMs interaction in a Parkin/Pink1-dependent manner, which could be considered a positive feedback response to ensure moderation of ER-mitochondria communication (Figure 5(a)) [98]. In DKD, both Parkin and Pink1 expressions are downregulated in experimental models of DKD and restoration of Parkin/Pink1 attenuates diabetic renal injury [99, 100]. This expression profile of Parkin/Pink1 is consistent with the finding of decreased MAMs formations in tubules observed in DKD [100]. Additionally, timely initiation of mitophagy represents an important quality control mechanism for cellular survival and repair during acute kidney injury (AKI) [101]. When considered together, it is tempting to hypothesize that manipulation of the Parkin/Pink1 expression enhances MAMs formation and promotes mitophagy, therefore preventing the mitochondrial dysfunction and progression of diabetic and acute kidney diseases.

#### 4.2.2. FUNDC1-Dependent Mitophagy

The adaptor proteins FUNDC1, BNIP3, and Nix harbor an LIR, which allows these proteins to dock LC3 to the OMM, and they have therefore been shown to be involved in mitophagy under hypoxic conditions. One recent study provided compelling evidence that FUNDC1 targets MAMs to mediate mitophagy [85]. FUNDC1 substantially translocates to MAMs in response to hypoxia by interacting with the ER resident protein calnexin. Next, it dissociates from calnexin and preferably recruits Drp1 to drive the mitochondrial fission essential for the engulfment of the mitochondria by autophagosomes (Figure 5(b)). Moreover, FUNDC1-dependent mitophagy has recently been shown to confer renoprotection in ischemic AKI through the correction of excessive mitochondrial fission [102]. The implications of these discoveries are pertinent to DKD, where the onset of hypoxia and mitochondrial fission have been demonstrated to occur in early-stage renal failure [82, 103], although the relevance of MAMs formation and function in this process in DKD is unknown. In addition to modulating mitophagy at MAMs, FUNDC1 promotes MAMs formation and ER Ca^2+^ release into mitochondria by binding to IP3Rs at a MAMs compartment in cardiocytes and podocytes [30, 50]. Indiscriminately increasing MAMs formation, therefore, would cause mitochondrial Ca^2+^ overload while promoting mitophagy, although it would gradually counteract the protective effect of mitophagy. A similar regulatory role of MAMs formation and its related effects on mitophagy and calcium homeostasis are observed with PACS2 [67, 104]. Hence, moderate and timely terminated increases in the formation of MAMs are a prerequisite to exerting their protective effects under conditions of stress. However, under DKD conditions, the expression of FUDNC1 was increased in glomeruli isolated from STZ-induced diabetic mice [50], while PACS2 was downregulated (our unpublished data). How and why these MAMs formation-related proteins underlying DKD pathology show opposite expression trends is not clear. The focus of future studies will involve demonstrating the overlapping and antagonistic roles of these two proteins in regulating MAMs formation, mitophagy, and mitochondrial Ca^2+^ in homeostasis in the settings of DKD.

### 4.3. Control of Mitochondrial Bioenergetics

Constitutive ER-to-mitochondria Ca^2+^ transfer through MAMs is essential for the maintenance of optimal mitochondrial bioenergetics under physiological conditions [105], because Ca^2+^ uptake by the mitochondria can facilitate the TCA cycle and oxidative phosphorylation at several sites, including at ATP synthase [106], *α*-ketoglutarate dehydrogenase (KGDH), isocitrate dehydrogenase (IDH), and pyruvate dehydrogenase (PDH) [107]. Thus, it is plausible that a moderate and timely increase in the Ca^2+^ level of the mitochondrial matrix due to enhanced MAMs communication can help the mitochondria to adapt to stress conditions that require more metabolic output. However, sustained Ca^2+^ transfer would lead to mitochondrial dysfunction [67], featuring increased mitochondrial ROS (mtROS) production, lower mitochondrial membrane potential (MMP), and decreased basal oxygen consumption. Recently, a study from Wei et al. addressed this pathway in podocytes within the setting of diabetes [50]. They demonstrated that diabetes enhanced the formation of MAMs in podocytes, which leads to more Ca^2+^ transport from the ER through the MAMs, resulting in elevated mitochondrial Ca^2+^ and consequently the mitochondrial dysfunction and the occurrence of glomerular damage [50]. Additionally, despite the high energy demand and abundant mitochondria content that make proximal tubular epithelial cells (PTECs) very vulnerable to mitochondrial dysfunction in DKD, no study has yet verified this scenario in tubular cells, especially in the PTECs. However, our recent study reached an opposing conclusion regarding MAMs formation, in which we found that MAMs formation was decreased in the tubules of patients and mice with DKD, and maintaining MAMs integrity via the DsbA-L/Mfn2 axis reduced tubular apoptosis, which also supported a protethering role of Mfn2 [74]. One plausible interpretation for this conflicting finding is that reduced MAMs at the latter stage of DKD would be detrimental to tubular survival, considering initially appropriated MAMs increase boosts in mitochondrial respiration and bioenergetics. Therefore, whether abnormal Ca^2+^ transport from the ER to mitochondria may occur in PTECs with a detrimental effect on mitochondrial function, especially at the early phase of DKD, will be a focus of future investigation. Nevertheless, our study still sheds some light on the impact of enhanced MAMs interaction on mitochondrial function. Additional studies are required to elucidate the kinetics and dynamic effects of Ca^2+^ transport from the ER to mitochondria on mitochondrial function during the progression of diabetic pathogenesis.

## 5. Regulation of Oxidative Stress

Normally, ROS are produced at physical levels that are necessary to maintain cellular homeostasis. Excessive ROS production, however, may induce oxidative damage to proteins, lipids, and DNA, ultimately leading to diabetic renal injury [108, 109]. mtROS overproduction contributes to many features of diabetic renal damage; a major site for mtROS production is the mitochondria respiratory chain where the electrons cannot be efficiently coupled by mitochondrial respiratory complexes I and III [110, 111]. However, other scenarios have also been suggested to account for this process.

First, excessive Ca^2+^ transfer via MAMs has been proposed to promote mtROS generation, and this hypothesis is supported by some evidence. For instance, diabetes induction augmented MAMs formation in podocytes led to elevated Ca^2+^ transfer from the ER to mitochondria, resulting in mtROS overgeneration. Suppressing MAMs formation by reducing FUNDC1, however, alleviated this effect [50]. These data link alterations in MAMs to mtROS excess in the pathogenesis of DKD. Furthermore, several regulators of Ca^2+^ channels found in MAMs might modulate Ca^2+^- and MAMs-dependent mtROS generation. For instance, Ero1*α* and Erp44, representative oxidoreductases of the ER, are highly enriched on MAMs [112]. At the molecular level, Ero1-*α* oxidizes IP3R1, causing the dissociation of ERp44 from IP3R1, which potentiates the Ca^2+^ transfer from the ER to mitochondria, resulting in mtROS overproduction. This event, in turn, could further promote Ca^2+^ signaling at MAMs through the Ero1*α*-dependent mechanism [113, 114], thereby forming a vicious cycle. However, the role of MAMs-associated Ero1-*α* has not been identified in diabetic kidney injury and thus more research is required to provide evidence to support this hypothesis.

Second, MAMs could directly produce mtROS via DsbA-L and p66Shc. DsbA-L, a multifunctional protein, has been found in the following locations in renal tubules [74, 115]: (a) the mitochondrial matrix, (b) the ER, and (c) the MAMs fraction. Furthermore, lower expression of DsbA-L is closely associated with increased mtROS production in tubular cells in DKD [116]. Whether DsbA-L modulates mtROS production in a MAMs-dependent manner, however, remains unclear. Regarding p66Shc, its positive regulatory roles in mtROS production and mitochondrial fission have been well documented in DKD [80, 117, 118], also by our groups. This effect is largely dependent on its phosphorylation at Ser36. It is noteworthy that p66Shc Ser36 phosphorylation also initiates the translocation of p66Shc to the MAMs fraction, where it could participate in mtROS production [119]. Further in-depth analysis found that the translocation of p66Shc to the MAMs is age dependent and corresponds well to mtROS production. Overall, these findings suggest that MAMs are a key player in preserving the mitochondrial redox state and, consequently, in regulating cellular redox balance. However, the detailed functional effect of MAMs on ROS overproduction is incompletely understood and little is known about its function in the occurrence and progression of diabetic oxidative renal injury.

## 6. Maintenance of Mitochondrial Calcium Homeostasis

The ER and mitochondria are the main organelles that regulate intracellular calcium (Ca^2+^) homeostasis, and it has been well established that Ca^2+^ is transferred from the ER lumen into the mitochondria through MAMs, which is essential for many aspects of cellular function. For instance, transient mitochondrial Ca^2+^ uptake via MAMs boosts mitochondrial oxidative respiration and ATP production by stimulating the rate-limiting enzymes of TCA cycles [120] and ATP synthase [121]. In addition to these effects, excessive Ca^2+^ uptake into the mitochondrial matrix can lead to mitochondrial Ca^2+^ overload and trigger the mitochondrial pathway of apoptosis by increasing the opening of the mPTP and the release of proapoptotic factors such as cytochrome c, caspase activation, and apoptosis in a plethora of cell types [122–125]. Furthermore, mitochondria calcium level is more sensitive to changes in ER Ca^2+^ dynamics rather than to the global Ca^2+^ signal [7, 8], emphasizing that the MAMs-mediated calcium communication between the ER and mitochondria plays a critical role in maintaining mitochondrial function and cellular homeostasis.

Structurally, the macromolecular complex allowing for Ca^2+^ transfer from the ER to mitochondria at the site of MAMs is basically composed of IP3R/Grp75/VDAC1. Grp75 is a molecular chaperone that physically interacts with IP3R and VDAC1 [25]. Upon cellular/stress stimulation, the Ca^2+^ released from the ER forms Ca^2+^ hotspots at the ER-mitochondria interface, is transported through the mitochondrial intermembrane space, and then finally enters the mitochondrial matrix via the mitochondrial calcium uniporter (MCU) [125]. Some researchers therefore refer to the “IP3R-Grp75-VDAC1-MCU” as the intracellular calcium regulation axis based on the route of Ca^2+^ transfer [126].

IP3R is the Ca^2+^ release channel located at the ER membrane; it has three isoforms, designated as type 1, type 2, and type 3. Immunochemistry studies have shown that IP3R isoforms were expressed in a cell-specific manner in the rat kidney [127]. More specifically, type 1 and type 3 were detected in mesangial cells and vascular smooth muscle cells and the latter was also observed in the principal cells of the cortical collecting ducts. Type 2 was expressed exclusively in the intercalated cells of collecting ducts. Interestingly, proximal tubular epithelial cells, constituting more than one-half of renal mass, expressed none of the isoforms of IP3R in the rat kidney. The IP3R protein expression profile in the human kidney has found to be drastically different through review of the publicly available data from the Human Protein Atlas. IP3R2 is the most abundant isoform type in the normal human kidney, particularly in the tubules, whereas IP3R1 and IP3R3 are expressed at very low levels in the glomeruli and tubules. It should be noted that all three IP3R isoforms have been reported to be involved in MAMs formation [19, 35, 128], whereas the IP3R1 and IP3R3 isoforms are expressed at very low levels in the kidney, at least in humans and mice, based on our previous study and the data from the Human Protein Atlas. In light of these findings, we speculated that IP3R2 is the main isoform that participates in the assembly of the Ca^2+^ channel complex at the MAMs. Additionally, despite some research related to MAMs that has been performed in podocytes, the type of IP3R that participates in MAMs formation in the podocytes remains unclear because these studies used a pan-antibody of IP3R to detect the expression of IP3R in the kidney. Therefore, using type-specific monoclonal antibodies to characterize their individual contributions to MAMs formation will be an important future priority, as a clear expression profile of IP3Rs in the kidney would facilitate a more accurate quantitative analysis of MAMs formation by PLA assay.

Regarding VDACs, the mammalian VDAC gene family consists of three isoforms, each of which shares approximately 70% sequence identity with the other two family members [129], and VDAC1 is the best known among VDAC isoforms [130]. To date, VDAC1 and VDAC2, located at the OMM, have been reported to support mitochondrial Ca^2+^ transfer from the ER and sarcoplasmic reticulum (SR), respectively [25, 131]. Findings from our previous studies noted that VDAC1 was involved in the intracellular calcium regulation axis in tubular cells in mice and patients with DKD, and similar results were observed in podocytes in diabetes- and Adriamycin-induced kidney diseases [50, 126]. However, no study has yet validated whether VDAC2/3 mediates the coupling of the ER and mitochondrial Ca^2+^ channels at the sites of MAMs, and therefore, further investigation is required.

In addition to Grp75, recent studies have shed light on other molecular partners at the MAMs such as RTN-1C and CypD, which regulate ER-mitochondria Ca^2+^ crosstalk. CypD is a Ca^2+^-sensitive mitochondrial chaperone that interacts with the VDAC1/Grp75/IP3R complex at the interface of MAMs and controls the Ca^2+^ transfer from the ER to mitochondria in the liver and heart through IP3R1 and IP3R2, respectively [35, 132]. Pharmacological and genetic inhibition of CypD reduced MAMs interactions, inhibited interorganellar Ca^2+^ exchange, and induced ER stress in the liver [132]. Notably, one recent study demonstrated that a lack of CypD seemingly aggravated renal structural damage in STZ-induced diabetic mice [133]. This damage might be associated with the reduced MAMs accompanied by the inhibition of CypD, which interferes with basic interorganellar Ca^2+^ exchange and subsequently induces ER stress in DKD.

Currently, little is known about the roles of MAMs-mediated Ca^2+^ communication in the pathogenesis of DKD. In a recent study [50], Wei and colleagues reported that the augmented formation of MAMs in podocytes due to hyperglycemic status is a critical step leading to mitochondrial calcium overload and damage of the glomerular structure and function in STZ-induced and db/db diabetic mice. Mechanistically, activation of TRPV1 by capsaicin reduced MAMs-mediated mitochondrial Ca^2+^ overload and dysfunction by repressing AMPK/FUNDC1-mediated MAMs formation in podocytes, suggesting that decreased MAMs formation in podocytes alleviates mitochondrial Ca^2+^ overload-induced mitochondrial dysfunction in the settings of diabetes. In this regard, inhibiting the excessive formation of MAMs may be an effective treatment against diabetic glomerulopathy. In future studies, it will be interesting to assess if podocyte-specific manipulation of mitochondrial Ca^2+^ uptake, for instance via MCU deletion, may have a similar effect on the progression of DKD.

Aside from mitochondrial dysfunction, uncontrolled mitochondrial Ca^2+^ accumulation, driven by increased MAMs formation, is detrimental to cell viability. In a previous report [126], antagonists against the calcium regulation axis reduced Ca^2+^ transfer through MAMs and prevented mitochondrial Ca^2+^ overload and subsequent podocyte apoptosis in an Adriamycin nephropathy rat model. Based on this report, we questioned whether prolonged or excessive Ca^2+^ transfer via MAMs may serve as an important mechanism of apoptosis in podocytes and tubular cells in DKD, although more work is needed to verify this hypothesis. Importantly, it should be noted that calcium uptake by mitochondria not only modifies mitochondrial function but also causes alterations in cytosolic Ca^2+^ activity. It is thus tempting to speculate that this abnormal uptake may affect cellular function, yet the exact consequences of this aberrant Ca^2+^ activity on renal injury require clarification. Nevertheless, these findings signal the possibility of maintaining mitochondrial Ca^2+^ balance by downregulating Ca^2+^-handling molecules in MAMs as a feasible strategy to prevent the propagation of dangerous signals in mitochondrial dysfunction- and apoptosis-related kidney diseases.

## 7. Involvement in Apoptosis

Apoptosis, a type of programmed cell death executed by caspases and resulting in the cleavage of cellular structural components [134], is diffusely increased and observed in the glomeruli, tubuli, and vascular endothelia in both the murine model and patients with DKD [135–140]. Both glomerular and tubular apoptosis predicted the loss of renal function, and furthermore, the acceleration of apoptotic processes in the glomeruli and tubules likely contributes to decreased nephron remodeling in patients with DKD [138]. Therefore, relieving cell death by preventing apoptosis is vital to reducing renal function decline. Recent data highlight that the MAMs platform is a critical hub for apoptosis in a variety of diseases, including DKD [15, 141]. Previously, we showed that MAMs formation, as evidenced by PLA staining and TEM analysis, was significantly reduced in the tubules of the diabetic kidney, which correlated with the extent of renal injury both in mice and patients with DKD [74]. Importantly, we also observed a close correlation between the loss of MAMs integrity and an increased number of apoptotic tubular cells in diabetes. These alterations were further exacerbated in diabetic DsbA-L^−/−^ mice. In vitro, the overexpression of DsbA-L in HK-2 cells restored MAMs integrity and reduced HG-induced apoptosis. These beneficial effects were partially blocked by the overexpression of FATE-1, a MAMs uncoupling protein, suggesting that maintenance of MAMs integrity in the tubules of the kidney exerts an antiapoptotic effect on tubular damage in diabetes.

In addition, PACS2, a multifunctional sorting protein, is involved in MAMs formation and interorganelle communication. Specifically, PACS2 links ER-mitochondria communication to mitochondria-dependent cellular apoptosis. When stimulated by apoptotic inducers, PACS2 could translocate Bid to the mitochondria to trigger the formation of mitochondrial truncated Bid (tBid) and initiate the intrinsic apoptotic pathway by inducing the formation of tBid and the release of cytochrome c, thereby causing cell death [15]. Notably, apoptosis is triggered by two main pathways: the intrinsic (mitochondria-dependent) and extrinsic (death receptor-dependent) pathways, and both apoptotic pathways are involved in renal cell apoptosis in DKD [142]. However, it seems that the intrinsic pathway is the main apoptotic pathway responsible for renal cell apoptosis, at least in tubular cells, based on our previous study and succinct studies from independent laboratories [80, 99, 143–145]. Additionally, our preliminary data showed that PACS2 was predominantly expressed in the tubular compartment whereas it was quite low in the glomerular compartment. In view of these findings, we hypothesize that PACS2-mediated MAMs formation is involved in the mitochondrial-dependent apoptotic pathway in tubular injury in DKD. Future studies will confirm whether PACS2 facilitates mitochondrial-dependent tubular cell apoptosis through MAMs-dependent mechanisms.

## 8. MAMs and Inflammation

Systemic and local low-grade inflammation and release of proinflammatory cytokines are implicated in the development and progression of DKD [146]. Inflammasomes are molecular platforms activated upon infection or cellular stress that trigger the maturation of proinflammatory cytokines such as IL-1*β* and IL-18 to engage immune defenses [147]. There are four subfamilies of inflammasomes depending on the sensor molecule: the NOD-like receptor family, pyrin domain-containing protein 3 (NLRP3), NOD-like receptor family, pyrin domain-containing protein 1 (NLRP1), NLR family, CARD domain containing 4 (NLRC4), and absent in melanoma 2 (AIM2) [147]. NLRP3 is ubiquitously expressed, although in the kidney, it is predominantly expressed in the tubules [148]. Our previous study demonstrated that chronic hyperglycemia promotes the activation of NLRP3 and NLRC4 inflammasomes, which contribute to the tubular damage in DKD [148, 149].

Notably, a recent study has provided some new insights into the interactive mechanisms of NLRP3 inflammasome formation and MAMs [10]. It is known that resting NLRP3 localizes to the ER membrane, but when activated, both NLRP3 and its adaptor redistribute to the ER-mitochondrial interface [10]. To date, the NLRP3 complex is the only inflammasome complex that has been associated with MAMs. Additionally, unlike other NLR family members, NLRP3 recognizes not only foreign pathogens but also danger-associated molecular patterns (DAMPs) released from damaged cells such as mtROS, mitochondrial DNA (mtDNA), and Ca^2+^ signaling [150]. In addition, Ca^2+^ can also activate NLRP3 inflammasomes in both direct and indirect ways, either through the intracellular Ca^2+^ directly binding to the calcium sensing receptor (CASR) and subsequently promoting NLRP3 activation [151] or through the sustained Ca^2+^ influx via MAMs to the mitochondrial matrix, which has been reported to trigger mPTP opening and thereby the release of DAMPs (i.e., mtROS and mtDNA) that trigger the activation of NLRP3 inflammasomes [10]. Recently, a delicate work demonstrated that increased MAMs formation promoted Ca^2+^ transfer via the MAMs platform in the podocytes in DKD [50]. Therefore, Ca^2+^ communication between the ER and the mitochondria might link the MAMs to NLRP3 inflammasome activation in DKD. More research is needed to verify this hypothesis.

A previous study indicated that NLRP3 inflammasome activity is negatively regulated by mitophagy [152], since mitophagy sweeps damaged mitochondria, thus protecting cells against the overgeneration of mtROS and mtDNA release and preventing NLRP3 inflammasome activation [10, 153]. Moreover, MAMs are also responsible for mitophagy activation as previously described, meaning that MAMs might regulate NLRP3 inflammasome activation by mitophagy. This theory is also supported by a study by Chen et al., in which they showed that enhanced mitophagy could inhibit the NLRP3 inflammasome activation of renal tubular cells in DKD [154]. Overall, the preceding information validates the permissive role of mitochondria in tubular injury, which acts as an upstream mediator of NLRP3 activation via cooperation with MAMs. Nevertheless, the detailed functional role of MAMs in inflammation in the setting of DKD is incompletely understood. Building upon these observations, further work is needed to explore the related influences and mechanisms by which MAMs modulate the inflammatory response.

## 9. MAMs and ER Stress

Disruption of ER homeostasis leads to the accumulation of unfolded or misfolded proteins, resulting in ER stress and triggering the unfolded protein response (UPR) [155]. Growing evidence has shown that ER stress contributes to the development and progression of DKD [6]. The adaptive phase of the UPR is initiated to restore homeostasis and protect cells against injury by facilitating ER degradation of the misfolded protein; excessively prolonged ER stress, however, can eventually cause cellular damage in diabetic kidneys [6].

Consistent with this finding, a previous report demonstrated that tunicamycin-induced ER stress resulted in more MAMs formation in the liver and muscle [67], but the rescue effect of increased MAMs formation on ER stress is time dependent. More specifically, during the early stage of ER stress, short-term MAMs formation increases contributed favorably to the cellular adaptation to ER stress by promoting Ca^2+^ transfer from the ER to the mitochondria matrix and finally boosting mitochondrial bioenergetics and ATP generation. With the assistance of a sufficient ATP supply, the ER exerts an optimal protein folding process by facilitating ER clearance of misfolded proteins. In contrast, the chronic enrichment of MAMs formation resulted in a slow and sustained increase in Ca^2+^ levels in the mitochondrial matrix and subsequently mitochondrial calcium overload-induced mitochondrial dysfunction in hepatocytes under obese condition [67]. Based on these findings, we could speculate that under prolonged ER stress conditions, as what happens in DKD, mitochondrial calcium overload mediated by increased MAMs would result. Therefore, careful fine-tuning of MAMs formation should be considered when working to alleviate ER stress in DKD. In addition, our recent study found that DsbA-L protected against DKD injury in a MAMs-dependent manner through two independent mechanisms: (1) by increasing MAMs formation so that ER stress was alleviated and (2) by promoting mitochondrial fusion, which restored mitochondrial agility and ultimately enabled a sufficient ATP supply. Thus, manipulation of the DsbA-L expression would ameliorate renal damage through maintaining MAMs integrity in DKD.

Interestingly, MAMs are not the only downstream effector of ER stress; MAMs dysfunction may in turn result in a disruption of interorganellar Ca^2+^ transfer and subsequent ER stress [15, 132]. RTNs are expressed predominantly in the ER membrane and produces three isoforms (i.e., RTN-1A, RTN-1B, and RTN-1C) that are involved in the regulation of ER stress [156]. Of these, RTN-1C has been previously demonstrated to be presented in the MAMs fraction. RTN-1C overexpressing cells showed an increase in MAMs and a decrease in mitochondrial Ca^2+^ uptake [37]. Combined with the results of a previous study showing that inhibition of interorganellar Ca^2+^ exchange induced ER stress, it could be speculated that the observed ER stress, related to the RTN-1C protein, could be attributed to the impaired ER-mitochondria Ca^2+^ transfer. Among three isoforms of RTN-1, only RTN-1A expression was more pronounced in patients with DKD, and it was stronger in the tubular compartment. RTN-1A has recently been identified as a new biomarker and therapeutic target for chronic kidney disease (CKD), including DKD, by inducing ER stress [156]. However, whether RTN-1A is a key component of MAMs like RTN-1C remains unclear. Further experiments are required to dissect the above associations and validate whether/how these molecular components (RTN1A/C) are involved in this process.

## 10. Conclusion

**Figure 6:**
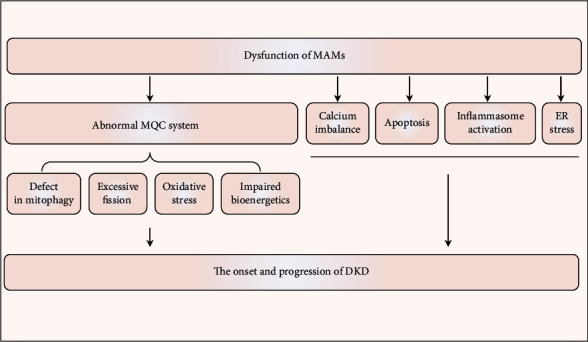


**Figure 7:**
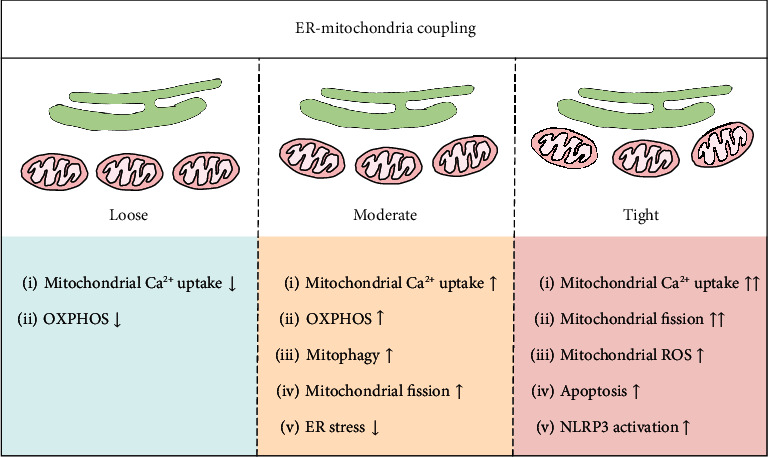


The importance of the miscommunication between the ER and mitochondria in the pathogenesis of kidney disease is gradually being recognized. The effect of MAMs' dysfunction on DKD is likely to result from the engagement of multiple pathways, including impaired mitochondrial quality control, abnormal Ca^2+^ flux, apoptosis, inflammation, and ER stress (Figure 6), despite limited currently available evidence for the precise role of MAMs in regulating diabetes-induced inflammasome activation, ER stress, and an abnormal MQC system in the kidney. Additionally, careful examination of the kinetics and dynamics of MAMs disruption in the progression of DKD urgently needs to be addressed. Since MAMs are highly plastic structures, the structural parameters (i.e., the number, length, and width of the MAMs) and the spatial-temporal pattern of MAMs are important aspects that determine their cellular function. In summary, under pathological conditions, such as during metabolic stress, a moderate and timely increase in MAMs ensures the propagation of IP3R-linked Ca^2+^ signals to the mitochondria to coordinate ATP production and alleviates ER stress and simultaneously promotes mitophagy by triggering mitochondrial fission. However, relaxing ER-mitochondria coupling suppresses the Ca^2+^ signal propagation to the mitochondria, putting at risk the Ca^2+^-dependent control of mitochondrial metabolism. In contrast, if MAMs couplings are too tight and widespread over a prolonged period of time, the initially beneficial effects for cellular function could convert to dangerous stimuli, including mitochondrial Ca^2+^ overload, malignant mitochondrial fission, inhibition of protective mitophagy, and activation of apoptosis and inflammasomes (Figure 7). In this regard, fine tuning of MAMs formation should be considered when developing it as a therapeutic target for diabetic renal injury.

## Figures and Tables

**Table 1:** Summary of the studies of MAMs' involvement in kidney disease.

Mouse model	Cell types	Changes in MAMs	Kidney outcomes	Ref.
Adriamycin-induced nephropathy	Podocytes	Increased	Promoting podocyte apoptosis and increasing proteinuria	[126]
Streptozocin- (STZ-) induced diabetic mice	Tubular cells	Decreased	Promoting tubule apoptosis and accelerating kidney damage	[74]
Db/db mice	Podocytes	Increased	Promoting mitochondrial Ca^2+^ overload and dysfunction, and exacerbating renal damage	[50]

**Table 2:** Summary of the functional roles of MAMs-resident proteins listed in this text.

Proteins	Relevant function(s) in MAMs	Expression in human kidney∗	
		Glomeruli	Tubules
(1) Protethering proteins			
PACS2	Regulation of apoptosis, ER homeostasis, Ca^2+^ between mitochondria and ER [15], and mitophagosome formation [104]	Low	Low
Grp75	Cytosolic molecular chaperone that links ER-resident IP3Rs to OMM-resident VDACs, resulting in increased MAMs formation and enhanced mitochondria Ca^2+^ uptake [25]	~High	High
IP3Rs	Interaction with Grp75 and VDACs, forming an intracellular calcium regulation axis in MAMs [25]	~medium	High
VDACs	Interaction with Grp75 and IP3Rs, forming an intracellular Ca^2+^ regulation axis in MAMs [25]	~high	High
Mfn2	Major modulator of ER-mitochondria tethering and mitochondrial fusion [26], but its role in organelle tethering is actually highly controversial, regardless of which tissue is considered. For a detailed discussion, please see the recent review in [157]	~Low	High
Mfn1	Tethering mitochondria to MAMs via interaction with ER-resident Mfn2 [26].	~Low	High
Fis1	Regulator of ER-mitochondria tethering via interaction with BAP31 and establishing a platform for apoptosis induction [27]. Fis1 dynamically regulates STX17 distribution at MAMs microdomains and induces Parkin-independent mitophagy [158]	High	High
BAP31	Interaction with TOM40 within MAMs and regulating mitochondrial function [159], as an interacting partner of CDIP1 and establishes an ER-mitochondrial crosstalk for ER stress-mediated apoptosis signaling [160]	~Medium	Medium~high
VAPB	Interacts with PTPIP51 to form a tethering complex [28, 29] and regulates autophagy by facilitating ER-mitochondria Ca^2+^ exchange [29]	Medium~high	Medium~high
PTPIP51	Interacts with the ER-resident protein VAPB to regulate ER-mitochondria associations and cellular Ca^2+^ homeostasis [28]. Interacting with ORP5/8 within MAMs to modulate mitochondrial morphology and function [161]	Medium	High
BECN1	Relocalization to the MAMs compartment in a Pink1-dependent manner and thereby enhances the formation of MAMs and autophagosomes [13]	Medium	Medium
FUNDC1	Localized to MAMs by binding to calnexin, where it promotes Drp1-dependent mitochondrial fission and mitophagy [162]. Additionally, binding of FUNDC1 to IP3R2 at the MAMs also increases the Ca^2+^ concentration in both the cytosol and the mitochondrial matrix, therefor promoting Fis1-dependent mitochondrial fission and mitophagy [30]	Low	Medium
PDZD8	Necessary for the formation of MAMs and required for mitochondrial Ca^2+^ uptake [31]	Medium	Medium
MITOL	Regulates mitochondrial dynamics and MAMs formation in a Mfn2-dependent manner [33] and maintains ER-mitochondria phospholipids transfer, such as in cardiolipin biogenesis [163]	NA	NA
Parkin	The role of Parkin in maintaining MAMs integrity is controversial. Some research indicates that Parkin tethers mitochondria to the ER by ubiquitination of Mfn2 [94, 95], while other results suggest that Parkin-mediated ubiquitination coupled to Pink1-catalyzed phosphorylation of Mfn2 dissociates mitochondria from the ER [164]	Medium	High

(2) IP3Rs/Grp75/VDAC complex-modulated proteins			
Sig-1R	Interacts with BiP and prolongs Ca^2+^ signaling from the ER into mitochondria by stabilizing IP3R3 at MAMs; increased Sig-1R in cells counteracts the ER stress response, whereas decreased Sig-1R enhances apoptosis [34]	~Low	Low~medium
CypD	Interacts with the VDAC1/Grp75/IP3R1 complex within MAMs and controls the Ca^2+^ transfer from the ER to mitochondria through IP3R1 [35]	NA	NA
Tespa1	Involved in maintaining MAMs integrity and functions as a regulator of mitochondrial Ca^2+^ flux from ER through the physical association with IP3R3 and GRP75, but not with VDAC1 [36]	Not detected	Medium
RTN-1C	Involved in maintaining MAMs integrity by binding to VDAC and FACL4 and affects mitochondrial morphology, Ca^2+^ responses, and lipid exchange with the ER [37]	Not detected	Not detected
GSK3*β*	GSK3*β* specifically interacts with the IP3R1/Grp75/VDAC1 complex in MAMs, and inhibition of GSK3*β* reduced both IP3R1 phosphorylation and ER Ca^2+^ release, which consequently diminishes both cytosolic and mitochondrial Ca^2+^ concentrations, as well as sensitivity to apoptosis [38]	~Low	~Medium
DISC1	Interacts with IP3R1 at MAMs and downregulates its ligand binding, thereby reducing the ER-mitochondria Ca^2+^ transfer [39]	Not detected	Medium
TOM70	Clusters at ER-mitochondria contacts, recruits IP3R3, and promotes ER to mitochondria Ca^2+^ transfer, bioenergetics, and cell proliferation [40]	Low	Medium
TGM2	TGM2 interacts with Grp75 within MAMs and regulates the interaction between IP3R3 and Grp75; the resulting association controls ER-mitochondrial Ca^2+^ flux and the profile of the MAMs proteome [41]	Medium	Medium
WFS1	Binds to NCS1 to form a complex with IP3R1 to activate ER-mitochondria Ca^2+^ crosstalk to promote mitochondrial function, i.e., activation of the TCA cycle and mitochondrial respiratory chain [42]	Low	High
PDK4	Moderates Ca^2+^ transfer from ER to mitochondria by interacting with and stabilizing the IP3R1-Grp75-VDAC1 complex at the MAMs\ interface and consequently maintains mitochondrial function [43]	Low	High
EI24	Tethering ER to mitochondria through forming a quaternary complex with IP3R1/Grp75/VDAC1 and regulating autophagy flux [44]	Medium	Medium

(3) Antitethering proteins			
TpMs	The expression of TpMs leads to mitochondrial fragmentation and loosens tethering with ER in a Mfn2-dependent manner [45]	Low	Medium
FATE1	Acts as a negative regulator of MAMs via interacting with the ER chaperones and emerin (EMD) and the mitochondrial protein Mic60/mitofilin and antagonizes calcium-induced apoptosis by uncoupling ER and mitochondria [46]	Not detected	Not detected
CAV1	Negatively regulates the formation of MAMs and therefore impairs Ca^2+^ transfer via the MAMs platform and compensatory mitochondrial bioenergetics response to early ER stress [47]; contrary results regarding the role of CAV1 in MAMs integrity were concluded by ref. [21].	Medium	Not detected

(4) Upstream regulators of the formation of MAMs			
GSK3*β*	Serves as a regulator of MAMs formation by regulating the VAPB–PTPIP51 interaction [28].	~Low	~Medium
p38 MAPK	Phosphorylation of Gp78 at S538 by p38 MAPK inhibits MAMs formation and mitochondrial fusion by promoting degradation of Mfn1/2 [48]	Not detected	Medium
PKG	Involved in the regulation of MAMs integrity [49]	Medium	Low
FOXO1	Augments MAMs formation via induction of PDK4 overexpression and promotes mitochondrial Ca^2+^ accumulation, mitochondrial dysfunction, and ER stress [43]	Low	Low
PKA	Phosphorylation and alterations in organelle distribution of the Drp1, thereby enhancing ER-mitochondria communication [47]	~Medium	Medium~high
AMPK*α*	Activation of AMPK suppresses the formation and function of MAMs by reducing the transcription of FUNDC1 [50]. AMPK binds directly to the MAMs tether Mfn2 and are therefore involved in MAMs formation [32]	Low	Medium

Note. ^∗^These data were freely obtained from the Human Protein Atlas (http://www.proteinatlas.org) based on immunohistochemistry staining in normal human kidney samples. Abbreviations: NA: not applicable; STX17: Syntaxin 17; CDIP1: cell death-inducing p53 target 1; ORP5/8: oxysterol-binding protein-related proteins 5/8; BiP: 78 KDa glucose-regulated protein; NCS1: neuronal calcium sensor 1; Gp78: autocrine motility factor receptor; TCA cycle: tricarboxylic acid cycle.

## Data Availability

The data used to support the findings of this study are included within the article.
